# Supplementary material for: Interventions addressing family violence and mental illness or substance use in low- and middle-income countries: A systematic review
Source: Glob Ment Health (Camb). 2023 Oct 18;10:e71. doi: 10.1017/gmh.2023.62 (PMC10643256; doi:10.1017/gmh.2023.62)
Supplement: Mootz et al. supplementary material [file S2054425123000626sup001.docx]

**Supplementary Table 1**

*Search Terms*

| Pillar Word | Search Terms (Keywords & Mesh/heading Terms)  → keyword.ti OR keyword.ab ORmesh.sh |
| --- | --- |
| LMIC | 1. Developing countr$.ti OR Developing countr$.ab OR Developing World.ti OR Developing World.ab OR LMIC.ti OR LMIC.ab OR Low Income Countr$.ti OR Low Income Countr$.ab OR Low and Middle Income Countr$.ti OR Low and Middle Income Countr$.ab OR Middle Income countr$.ti OR Middle Income countr$.ab OR Third World Countr$.ti OR Third World Countr$.ab OR Underdeveloped Countr$.ti OR Underdeveloped Countr$.ab OR Developing countries.sh  2. (Africa or Asia or Caribbean or West Indies or South America or Latin America or Central America).ti,ab.  3. (Afghanistan or Albania or Algeria or Angola or Antigua or Barbuda or Argentina or Armenia or Armenian or Aruba or Azerbaijan or Bahrain or Bangladesh or Barbados or Benin or Byelarus or Byelorussian or Belarus or Belorussian or Belorussia or Belize or Bhutan or Bolivia or Bosnia or Herzegovina or Hercegovina or Botswana or Brasil or Brazil or Bulgaria or Burkina Faso or Burkina Fasso or Upper Volta or Burundi or Urundi or Cambodia or Khmer Republic or Kampuchea or Cameroon or Cameroons or Cameron or Camerons or Cape Verde or Central African Republic or Chad or Chile or China or Colombia or Comoros or Comoro Islands or Comores or Mayotte or Congo or Zaire or Costa Rica or Cote d'Ivoire or Ivory Coast or Croatia or Cuba or Cyprus or Czechoslovakia or Czech Republic or Slovakia or Slovak Republic or Djibouti or French Somaliland or Dominica or Dominican Republic or East Timor or East Timur or Timor Leste or Ecuador or Egypt or United Arab Republic or El Salvador or Eritrea or Estonia or Ethiopia or Fiji or Gabon or Gabonese Republic or Gambia or Gaza or Georgia Republic or Georgian Republic or Ghana or Gold Coast or Greece or Grenada or Guatemala or Guinea or Guam or Guiana or Guyana or Haiti or Honduras or Hungary or India or Maldives or Indonesia or Iran or Iraq or Isle of Man or Jamaica or Jordan or Kazakhstan or Kazakh or Kenya or Kiribati or Korea or Kosovo or Kyrgyzstan or Kirghizia or Kyrgyz Republic or Kirghiz or Kirgizstan or Lao PDR or Laos or Latvia or Lebanon or Lesotho or Basutoland or Liberia or Libya or Lithuania or Macedonia or Madagascar or Malagasy Republic or Malaysia or Malaya or Malay or Sabah or Sarawak or Malawi or Nyasaland or Mali or Malta or Marshall Islands or Mauritania or Mauritius or Agalega Islands or Mexico or Micronesia or Middle East or Moldova or Moldovia or Moldovian or Mongolia or Montenegro or Morocco or Ifni or Mozambique or Myanmar or Myanma or Burma or Namibia or Nepal or Netherlands Antilles or New Caledonia or Nicaragua or Niger or Nigeria or Northern Mariana Islands or Oman or Muscat or Pakistan or Palau or Palestine or Panama or Paraguay or Peru or Philippines or Philipines or Phillipines or Phillippines or Poland or Portugal or Puerto Rico or Romania or Rumania or Roumania or Russia or Russian or Rwanda or Ruanda or Saint Kitts or St Kitts or Nevis or Saint Lucia or St Lucia or Saint Vincent or St Vincent or Grenadines or Samoa or Samoan Islands or Navigator Island or Navigator Islands or Sao Tome or Saudi Arabia or Senegal or Serbia or Montenegro or Seychelles or Sierra Leone or Slovenia or Sri Lanka or Ceylon or Solomon Islands or Somalia or South Africa or Sudan or Suriname or Surinam or Swaziland or Syria or Tajikistan or Tadzhikistan or Tadjikistan or Tadzhik or Tanzania or Thailand or Togo or Togolese Republic or Tonga or Trinidad or Tobago or Tunisia or Turkey or Turkmenistan or Turkmen or Uganda or Ukraine or Uruguay or USSR or Soviet Union or Union of Soviet Socialist Republics or Uzbekistan or Uzbek or Vanuatu or New Hebrides or Venezuela or Vietnam or Viet Nam or West Bank or Yemen or Yugoslavia or Zambia or Zimbabwe or Rhodesia).ti,ab.  4. ((developing or less* developed or under developed or underdeveloped or middle income or low* income or underserved or under served or deprived or poor*) adj (countr* or nation? or population? or world)).ti,ab.  5. ((developing or less* developed or under developed or underdeveloped or middle income or low* income) adj (economy or economies)).ti,ab.  6. (low adj3 middle adj3 countr*).ti,ab.  7. (lmic or lmics or third world or lami countr*).ti,ab.  8. transitional countr*.ti,ab.  9. or/1-8 |
| Intervention | Acceptance and Commitment Therapy.ti OR Acceptance and Commitment Therapy.ab OR Behavior Therapy.ti OR Behavior Therapy.ab OR Clinical Trial$.ti OR Clinical Trial$.ab OR Alcoholics Anonymous.ti OR Alcoholics Anonymous.ab OR Clinical Study.ti OR Clinical Study.ab OR Cognitive Behavioral Therapy.ti OR Cognitive Behavioral Therapy.ab OR Cognitive Therapy.ti OR Cognitive Therapy.ab OR Community Counseling.ti OR Community Counseling.ab OR Community Mental Health.ti OR Community Mental Health.ab OR Community Mental Health Service$.ti OR Community Mental Health Service$.ab OR Community Psychiatry.ti OR Community Psychiatry.ab OR Community Psychology.ti OR Community Psychology.ab OR Counseling.ti OR Counseling.ab OR Counseling Psycholog$.ti OR Counseling Psycholog$.ab OR Couples Psychotherapy.ti OR Couples Psychotherapy.ab OR Crisis Intervention$.ti OR Crisis Intervention$.ab OR Dialectical Behavior Therapy.ti OR Dialectical Behavior Therapy.ab OR Domestic Abuse Intervention Project.ti OR Domestic Abuse Intervention Project.ab OR Duluth Model.ti OR Duluth Model.ab OR Early Intervention$.ti OR Early Intervention$.ab OR Family Therapy.ti OR Family Therapy.ab OR Group Counseling.ti OR Group Counseling.ab OR Group Psychotherapy.ti OR Group Psychotherapy.ab OR Interpersonal Therapy.ti OR Interpersonal Therapy.ab OR Intervention$.ti OR Intervention$.ab OR Intervention Study.ti OR Intervention Study.ab OR Mental Health Program$.ti OR Mental Health Program$.ab OR Mental Health Service$.ti OR Mental Health Service$.ab OR Mindfulness.ti OR Mindfulness.ab OR Motivational Interviewing.ti OR Motivational Interviewing.ab OR Narcotics Anonymous.ti OR Narcotics Anonymous.ab OR Narrative Therapy.ti OR Narrative Therapy.ab OR Randomized Clinical Trial$.ti OR Randomized Clinical Trial$.ab OR Randomized Control Trial$.ti OR Randomized Control Trial$.ab OR Randomized Controlled Trial$.ti OR Randomized Controlled Trial$.ab OR Psychoeducation.ti   OR Psychoeducation.ab OR Psychological Techniques.ti OR Psychological Techniques.ab OR Psychosocial Intervention$.ti OR Psychosocial Intervention$.ab OR Psychotherapeutic Outcome$.ti OR Psychotherapeutic Outcome$.ab OR Psychotherapeutic Techniques.ti OR Psychotherapeutic Techniques.ab OR Psychotherapy.ti OR Psychotherapy.ab OR Therapeutic Process$.ti OR Therapeutic Process$.ab OR Therapy.ti OR Therapy.ab OR Treatment$.ti OR Treatment$.ab OR intervention.sh OR mental health programs.sh OR treatment.sh OR Treatment Effectiveness Evaluation.sh OR Mental Health Program Evaluation.sh OR Treatment Outcomes.sh |
| Mental Illness/Substance Use | Affective Disorder$.ti OR Affective Disorder$.ab OR Affective Symptom$.ti OR Affective Symptom$.ab OR Anxiety.ti OR Anxiety.ab OR Anxiety Disorder$.ti OR Anxiety Disorder$.ab OR Behavior Disorder$.ti OR Behavior Disorder$.ab OR Depression.ti OR Depression.ab OR Dysthymic Disorder$.ti OR Dysthymic Disorder$.ab OR Major Depression.ti OR Major Depression.ab OR Mental Disorder$.ti OR Mental Disorder$.ab OR Mental Health.ti OR Mental Health.ab OR Mental Illness.ti OR Mental Illness.ab OR Neurosis.ti OR Neurosis.ab OR Neurotic Disorder$.ti OR Neurotic Disorder$.ab OR Post-traumatic stress disorder$.ti OR Post-traumatic stress disorder$.ab OR Post-Traumatic Stress.ti OR Post-Traumatic Stress.ab OR Posttraumatic Stress Disorder$.ti OR Posttraumatic Stress Disorder$.ab OR PTSD.ti OR PTSD.ab OR Stress Disorder$.ti OR Stress Disorder$.ab OR Suicid$.ti OR Suicid$.ab OR SUMIC Syndemic.ti OR SUMIC Syndemic.ab OR Syndemic.ti OR Syndemic.ab OR Sava Syndemic.ti OR Sava Syndemic.ab OR common mental disorder*.ti OR common mental disorder*.ab OR mental distress.ti OR mental distress.ab OR stress.ti OR stress.ab OR idioms of stress.ti OR idioms of stress.ab OR mental disorders.sh OR mental health.sh  OR  Alcohol$.ti OR Alcohol$.ab OR Alcohol addiction.ti OR Alcohol addiction.ab OR Alcohol consumption.ti OR Alcohol consumption.ab OR Alcohol dependenc$.ti OR Alcohol dependenc$.ab OR Alcohol disorder.ti OR Alcohol disorder.ab OR Cannabis.ti OR Cannabis.ab OR Cannabis addiction.ti OR Cannabis addiction.ab OR Cannabis dependenc$.ti OR Cannabis dependenc$.ab OR Cocaine.ti OR Cocaine.ab OR Cocaine addiction.ti OR Cocaine addiction.ab OR Cocaine dependenc$.ti OR Cocaine dependenc$.ab OR Crack addiction.ti OR Crack addiction.ab OR Crack dependenc$.ti OR Crack dependenc$.ab OR Drug$.ti OR Drug$.ab OR Drug Addiction.ti OR Drug Addiction.ab OR Drug Dependenc$.ti OR Drug Dependenc$.ab OR Heroin.ti OR Heroin.ab OR Heroin addiction.ti OR Heroin addiction.ab OR Heroin dependenc$.ti OR Heroin dependenc$.ab OR Khat.ti OR Khat.ab OR Khat addiction.ti OR Khat addiction.ab OR Khat dependenc$.ti OR Khat dependenc$.ab OR Marijuana.ti OR Marijuana.ab OR Marijuana addiction.ti OR Marijuana addiction.ab OR Marijuana dependenc$.ti OR Marijuana dependenc$.ab OR Meth.ti OR Meth.ab OR Meth addiction.ti OR Meth addiction.ab OR Meth dependenc$.ti OR Meth dependenc$.ab OR Methamphetamine.ti OR Methamphetamine.ab OR Methamphetamine dependenc$.ti OR Methamphetamine dependenc$.ab OR Methamphetamine addiction.ti OR Methamphetamine addiction.ab OR Narcotic$.ti OR Narcotic$.ab OR Narcotic addiction.ti OR Narcotic addiction.ab OR Narcotic dependenc$.ti OR Narcotic dependenc$.ab OR Opioids.ti OR Opioids.ab OR Opioid addiction.ti OR Opioid addiction.ab OR Opioid dependenc$.ti OR Opioid dependenc$.ab OR Stimulant.ti OR Stimulant.ab OR Substance Abuse.ti OR Substance Abuse.ab OR drug abuse.sh OR Alcoholism.sh OR Drug Addiction.sh OR “Substance Use Disorder”.sh |
| Family Violence | Child Abuse.ti OR Child Abuse.ab OR Domestic Violence.ti OR Domestic Violence.ab OR Family Conflict.ti OR Family Conflict.ab OR Familial Conflict.ti OR Familial Conflict.ab OR Family Functioning.ti OR Family Functioning.ab OR Family Violence.ti OR Family Violence.ab OR Household Violence.ti OR Household Violence.ab OR Interpersonal Violence.ti OR Interpersonal Violence.ab OR Marital Rape.ti OR Marital Rape.ab OR Partner Violence.ti OR Partner Violence.ab OR Domestic Violence.sh OR Family Conflict.sh OR Partner Abuse.sh |

**Supplementary Table 2**

*Characteristics of Included Studies and Interventions*

| **Authors, Year** | **Country** | **Design** | **Population** | **Sample Size** | **Intervention** | **Adapted** | **Format** | **Facilitators** | **Study Arms** |
| --- | --- | --- | --- | --- | --- | --- | --- | --- | --- |
| Buller et al., 2016;  Hidrobo et al., 2016 | Ecuador | Two-stage Cluster RCT | Low-income, married/partnered urban adolescent girls 15 years old and over and women | 1,226 | World Food Programme gave aid in food or money if a monthly nutrition group was attended | No* | Aid of $40 (about 11% of pre-intervention income) given once a month for 6 months | Two Quito women with experience interviewing vulnerable populations. | (1) cash; (2) food; (3) food vouchers; (4) control |
| Chaudhury et al., 2016;  Betancourt et al., 2017 | Rwanda | RCT | At least one school-aged child and adult, HIV-positive caregiver, willing to discuss HIV status with children in rural Rwanda | 293 (82 families) | Family Strengthening Intervention (FSI) for HIV identified family strengths and challenges, how HIV affects them, and how to improve communication. | Yes | 6 modules of about 90 minutes over 6 months with follow-up at three months | Three male and three female bachelor-level Rwandan counselors trained in FSI. TAU was through the Ministry of Health | (1) Family Strengthening Intervention for HIV; (2) Treatment as Usual (standard social work support only) |
| Cluver et al., 2016; 2018 | South Africa | Cluster RCT | Primary caregivers with an adolescent (10-18 years) from 40 peri-urban townships | 552 families- (552 caregivers and 548 adolescents) | Sinovuyu Teen: Building relationship, praise, emotion management, problem solving, conflict management, rules/routines, and money management | Locally developed | 14 sessions weekly (10 sessions jointly; 4 sessions separately) | Trained local community members, regional NGO staff member, local social auxiliary workers | (1) Sinovuyo Teen; (2) Control (1-day hygiene program) |
| Gilbert et al., 2017 | Kyrgyzstan | Pre/post design | Bishkek and Osh resident women with illicit drug use or binge drinking | 66 | Women Initiating New Goals of Safety (WINGS). IPV and GBV screening, brief intervention, and treatment referral with HIV counselling and testing. | Yes | 2 sessions | Non-government organization caseworkers | (1) WINGS group pre and post |
| Glass et al., 2017 | Democratic Republic of Congo | Randomized Community Trial | Adult women and men over age 16 from 10 rural villages in Eastern Democratic Republic of Congo, one per household | 833 | Pigs for Peace, a livestock productive asset transfer intervention that included a female piglet, training, and veterinarian services | Locally developed | Followed from 0-18 months | N/A | (1) Pigs for Peace; (2) delayed control |
| Gupta et al., 2013;  Annan et al., 2017 | Côte d'Ivoire | RCT | Female adults in rural Côte d'Ivoire without previous experience with a savings program | 934 | Village savings and loans association (VSLA) economic empowerment and savings program plus 8-session gender dialogue group (GDG) based on Stages of Change | No | Everyone met once a week for VSLA; treatment groups also met bi-weekly for GDG sessions (1.5-2.5 hours) over 4 months | Each group had one male and one female IRC field agent, one in GBV and one in economic recovery, trained in facilitation | (1) Village Savings and Loan Association and Gender Dialogue Group; (2) Village Savings and Loan Association only |
| Jewkes et al., 2006; 2008 | South Africa | Cluster RCT | Adolescents and adult men and women ages 16-23 with HIV from 70 rural villages | 2,776 | Stepping Stones (13 core lessons) improves sexual health through promotion of gender-equitable relationships and improved communication and education about various topics related to sexual health, sexually transmitted infections, gender-based violence, and relationships | Yes | Group: 3 hours per session, 17 sessions over 3-12 weeks | 11 facilitators (young men and women) with post-school qualification,  3 weeks of training | (1) Stepping Stones; (2) Control Group: 2-3-hr session on HIV and safer sex. |
| Jewkes et al., 2014 | South Africa | Pre/post, no control | Male and female younger adults (aged 18 to 34) in urban informal settlements | 232 | 10 sessions of Stepping Stones with 11 sessions of Creating Futures (livelihood empowerment) | Locally developed | 3-hour, single-sex, group sessions held 2x week over 12 weeks | Facilitators who had completed secondary school and some health sector experience | (1) Intervention |
| Jones et al., 2014 | Zambia | Quasi-experimental | Adult heterosexual couples in Lusaka (urban community health clinics) where at least one has HIV | 394 | The Partner Project focuses on reducing HIV risk with couples through psychoeducation about sexual risk prevention and training in negotiation and communication | Yes | 4 gender-specific group sessions | Partner Project research staff compared to non-specialized community health center (CHC) staff members | (1) Research staff-led intervention; (2) CHC-led intervention; (3) control group |
| Lachman et al., 2017 | South Africa | RCT | Low-income parent-child (3-8 years) dyads in Cape Town | 68 parent-child dyads | Sinovuyo Caring Families Program for Young Children to improve parent-child relationships | Locally developed | 3 parent groups (10-14 parents per group) 2-3 hrs weekly for 12 weeks | Community-based workers with a basic level of training in early childhood development | (1) Intervention; (2) waitlist control |
| L’Engle et al., 2014; Parcesepe et al., 2015; 2016 | Kenya | Multisite RCT | Adult female sex workers in Mombasa who were moderate-risk drinkers and negative for STIs except HIV | 818 | Based on WHO brief intervention for alcohol use, with elements of states of change and social cognitive health behavior change theories, adapted for Kenyan culture via focus groups | Yes | 1, 20-min session a month for 6 months | Nurse counselors trained in motivational interviewing | (1) 6-session alcohol harm reduction intervention; (2) Control, 6- session nutrition seminar |
| Meffert et al., 2011 | Egypt | RCT | Adult male & female Sudanese refugees with PTSD symptoms were recruited through preexisting community contacts | 22 | Interpersonal therapy (IPT); Waitlist control. | No* | Group: 2x/week for 3 weeks | Sudanese community therapists without previous mental health training | (1) IPT;  (2) waitlist control |
| Mutisya et al., 2018 | Kenya | QE | Pregnant adult women seeking antenatal care with positive gender-based violence scores | 283 | Psychosocial intervention on GBV, safety planning and referrals for GBV-services in area. Antenatal treatment as usual (TAU). | Yes | Individual: 3, 30-min sessions over a 5-month period. | Research assistants with experience in social work | (1) Psychosocial intervention; (2) antenatal Treatment as Usual |
| Nadkarni et al., 2017a,b | India | RCT | Male adults with harmful drinking behaviors (AUDIT: 12-19) recruited through public primary healthcare centers in Goa | 377 | Counseling for Alcohol Problems (CAP) uses a Motivational Interviewing approach and involves assessment and personalized feedback (initial phase), developing cognitive and behavioral skills (middle phase), and learning how to manage relapse (ending phase). | Locally developed | Delivered in three phases over a maximum of four sessions (30–45 min) at weekly to fortnightly intervals | Lay counselors | (1) Counseling for Alcohol Problems treatment and enhanced usual care; (2) Enhanced Usual Care |
| Patel et al., 2017; Weobong et al., 2017 | India | RCT | Male and female adults in primary care with moderately severe to severe depression | 495 | Healthy activity programme: focused on behavioral activation that includes psychoeducation, behavioral assessment, activity monitoring, structuring and scheduling, activation of social networks, and problem solving | Locally developed | Individual sessions (30-40 min) weekly over 6-8 weeks | Lay counselors with a high school education trained to administer the intervention | (1) Enhanced usual care with primary care physician; (2) Enhanced Usual Care with Healthy Activity Programme in randomly sized groups |
| Satyanarayana et al., 2016 | India | RCT | Male adult inpatients with alcohol dependency, married with a child under 16, perpetrated any form of IPV in past six months whose wife was primary caregiver (also collected wives’ mental health measures) | 177 | An integrated cognitive-behavioral intervention (ICBI) addressing the relationship between, triggers and consequences of IPV and alcohol | Locally developed | 8 individual 45 to 60-minute sessions | Master's degree in clinical psychology and certified in the intervention | (1) Integrated Cognitive Behavioral Intervention; (2) Treatment As Usual control of 1 session of psychoeducation and pharmacotherapy |
| Tankard et al., 2019 | Colombia | RCT | Female adults aged 18-55 with a male partner and interest in a savings account in four urban settings across Valle del Cauca | 1800 | Proyecto Crecer (Project Grow): Three health check-ups and a free savings account with 10,000 pesos ($5) and matching savings at a 1/3 ratio. | Locally developed | Accounts were opened and surveys occurred at baseline, 9 months, and 18 months | Female survey enumerators employed by a Colombian survey research firm | (1) savings account with health services; (2) health services |
| Tiwari et al., 2010 | China | RCT | Women in Hong Kong who have experienced IPV | 200 | The initial session focused on empowerment via safety planning. Phone calls acted as social support and a 24h access to a hotline. Aims were to increase safety and enhance problem solving and decision-making. | Yes | One in-person session (30 min) and 12 phone calls (15-20 min) once a week over 12 weeks | Trained research assistants (social workers) | (1) Advocacy intervention; (2) control |
| Witte et al., 2011; Carlson et al., 2012 | Mongolia | RCT | Adult female sex workers with harmful alcohol use | 166 | HIV Sexual Risk Reduction to increase knowledge and skills for safety. Enhancement included 2 sessions of Motivational Interviewing with a focus on alcohol use. | Yes* | 4 weekly 90-min group sessions | Mongol-speaking female facilitators | (1) HIV Sexual Risk Reduction; (2) HIV Sexual Risk Reduction + Motivational Interviewing; (3) Control wellness promotion |

**Design:** RCT= Randomized Control Trial; Cluster RCT= Cluster Randomized Control Trial; QE= quasi experimental design

**Adapted**: *=noted specifically as given in another language if not locally developed
